# Low Expression of GIGYF1 Inhibits Metastasis, Proliferation, and Promotes Apoptosis and Autophagy of Gastric Cancer Cells

**DOI:** 10.7150/ijms.82719

**Published:** 2023-06-12

**Authors:** Linqi Zhu, Zhendong Yao, Qingxin Luo, Yun Liu, Wenjun Zhao, Chen Shao, Shihe Shao, Feilun Cui

**Affiliations:** 1 The Affiliated People's Hospital of Jiangsu University. 212002 Zhenjiang, Jiangsu, China.; 2 The Affiliated Yixing Hospital of Jiangsu University. 214200 Wu Xi, Jiangsu, China.; 3 The Affiliated Hospital of Jiangsu University. 212008 Zhenjiang, Jiangsu, China.

**Keywords:** GIGYF1, gastric cancer, ERK, AKT

## Abstract

GRB10 interacting GYF protein 1 (GIGYF1) binds to the N-terminal region of Grb10, regulates multiple signaling pathways. However, it is not clear what happens to cell proliferation, metastasis, apoptosis, and autophagy when the expression level of GIGYF1 gene is reduced. Detection of GIGYF1 expression in clinical tissue specimens and gastric cancer (GC) cell lines by quantitative Real-time PCR (qRT-PCR), GIGYF1 gene was knocked down in MGC-803 cells using small interfering RNA, the effect of GIGYF1 gene on cell metastasis was detected using Transwell assay and wound healing assay, the effect on cell proliferation was detected using plate cloning assay and cck-8 assay, the effect on apoptosis was detected using flow cytometry, autophagosomes were detected using laser confocal microscopy, and the effect on protein expression was detected using immunofluorescence and Western blotting. GIGYF1 gene expression was higher in tumor tissue samples than in paracancer tissue samples, and higher in human GC cell lines than in human normal gastric epithelial cells. GIGYF1 gene knockdown inhibited cell migration, scratch healing ability and EMT process, weakened cell proliferation ability, increased apoptosis rate, promoted the formation of autophagosomes, and changed the corresponding protein expression level. Meanwhile, GIGYF1 knockdowns inhibited the ERK and AKT signaling. In conclusion, the low expression of GIGYF1 gene can inhibit the occurrence and progression of gastric cancer, during which the ERK and AKT signaling pathways are inhibited.

## Introduction

Gastric cancer (GC) is one of the most common gastrointestinal tumors, it is associated with various factors, such as environment, diet, lifestyle and genetic inheritance^1^. According to the latest global GC data, China has the highest incidence of GC^2^. Patients with GC usually show non-specific gastrointestinal symptoms in the early stage^3^. Symptoms, such as epigastric pain, decreased appetite, and painful swallowing, may appear with the malignant progression of the disease. The prognosis of GC is closely related to the clinical progress^4^,^5^. Even if the advanced GC has received comprehensive treatment mainly based on surgery, the 5-year survival rate is still less than 30%. While most of the early GC can receive radical treatment under endoscope, with the 5-year survival rate exceeding 90%, but the detection rate of early GC in China is less than 10%^6^-^7^,^8^. Therefore, it is necessary to find biomarkers with significant specificity and sensitivity for early diagnosis to improve the detection rate of early GC.

GIGYF1 gene is located in 7q22, with a total length of about 10kb, and can bind to the N-terminal region of Grb10. Current studies have proved that GIGYF1 can cooperate with Grb10 to regulate IGF-1R signaling pathway and affect diabetic encephalopathy, and is significantly expressed in myocardial tissue, which is of great significance for the occurrence and progression of congenital heart disease^9^,^10^,^11^,^12^. GIGYF1 can combine with AKT to form macromolecular complex, the AKT signaling pathway plays an important role in regulating cell apoptosis and autophagy^11^,^13^-^14^. When the expression level of GIGYF1 decreases, the expression level of p-AKT decreases, leading to cell apoptosis. Grb10 can promote the expression of downstream ERK1/2, and ERK1/2 can promote the transcription and translation process of cells^15^-^16^. Therefore, GIGYF1 may indirectly regulate cell growth and proliferation by binding with Grb10. However, these mechanisms have not been clearly verified, and it is not clear what changes the decreased expression level of GIGYF1 gene will cause on cell proliferation, migration, apoptosis and autophagy.

At present, no research has confirmed the role of GIGYF1 gene in GC. In this article, we mainly studied whether GIGYF1 gene can play an important role in GC cell metastasis, proliferation, apoptosis and autophagy.

## Materials and methods

### Reagents

Clinical tissue specimens were collected from Affiliated People's Hospital of Jiangsu University. The human normal gastric epithelial cells (GES-1) and human GC cell lines were purchased from Beijing Beina Chuanglian Biotechnology Research Institute. The RPMI 1640 medium was obtained from Gibco (USA). Fetal bovine serum was supplied by Hycone Inc (USA). Penicillin-streptomycin was acquired from Beijing Solarbio Company. The Annexin V-FITC Apoptosis Detection Kit was bought from Beyotime. GIGYF1 small interference RNA and control NC were purchased from Suzhou Jima Biotechnology Company. Lipofectamine 3000 was provided by Thermo Fisher Company (USA). Trizol reagent, reverse Transcription reagent was obtained from HiScript® III 1ST Strand cDNA Synthesis Kit (+gDNA WIper) AceQ qPCR SYBR Green Master Mix, and the ECL exposure solution was purchased from Vazyme Biotech Company. Cell Signaling Technology Company (USA) provided the rabbit Anti GAPDH antibody (dilution ratio was 1:10000) and the rabbit anti LC3B antibody (dilution ratio was 1:2000). Wanlei Biotechnology Company supplied the Hoechst 33342 stain. Proteintech (USA) provided the Rabbit Anti N-cadherin antibody (dilution ratio was 1:3000), rabbit anti-E-cadherin antibody (dilution ratio was 1:5000), rabbit anti-Vimentin antibody (dilution ratio was 1:3000), rabbit Anti-Snail Antibody (dilution ratio was 1:2000), rabbit Anti-Bax Antibody (dilution ratio was 1:3000), rabbit Anti-BCL-2 Antibody (dilution ratio was 1:3000), rabbit Anti-PCNA Antibody (dilution ratio was 1:3000), rabbit Anti-MMP2 Antibody (dilution ratio was 1:1000), rabbit Anti-p-ERK 1/2 Antibody (dilution ratio was 1:1000), rabbit Anti- ERK 1/2 Antibody (dilution ratio was 1:1000), rabbit Anti-p-AKT Antibody (dilution ratio was 1:1000), rabbit Anti-AKT Antibody (dilution ratio was 1:1000). Thermo Fisher Company (USA) supplied the Rabbit Anti-GIGYF1 Antibody (dilution ratio was 1:1000). Horseradish Peroxidase labeled Goat Anti-rabbit IgG HRP was purchased from UNIV Company **(**dilution ratio was 1:20000).

### Survival analysis

The Kaplan-Meier plotter (http://www.kmplot.com) database evaluated the association of the GIGYF1 mRNA expression level with the outcome in patients with GC. This database is constructed based on gene chips and RNA seq data from public databases such as GEP, EGA, and TCGA, and integrates genetic information and clinical prognostic value for survival analysis. According to the different quantile of GIGYF1 expression, the patients were divided into 246 patients with high expression and 385 patients with low expression. HR and logrank P values were calculated to evaluate the correlation between gene expression and survival.

### Tissue specimens

Informed consent was provided by the participants. Our study protocols gained approval from Ethics Committee of the School of Medicine, Jiangsu University. During the surgery, clinical doctors collected tissue samples, removed tumor tissue and paracancer tissue (1cm away from the tumor tissue). Fresh tumor tissue samples as well as paired paracancer tissue samples from 42 GC patients were collected for RNA and protein extraction. The GC samples were harvested in cases enrolled from the affiliated hospital of Jiangsu University. All participants were selected randomly, and no participant received chemotherapy or radio-therapy before surgery.

### Cell culture

The cells were cultured in RPMI 1640 medium containing 10% fetal bovine serum and 1% penicillin-streptomycin in an incubator at 37℃ and 5% CO_2_ for subsequent experiments.

### Real-time fluorescence quantitative PCR

The total RNA of the cells were extracted according to the instructions of the Trizol reagent, and the expression of GIGYF1 was calculated by the 2^-δδCt^ method with GAPDH as an internal reference. The primers of GIGYF1 upstream and downstream were 5'-AAGGGCTAGAGGAGGAAGGG-3' and 5'-GTATGTACGTCCCAGAGGCG-3', respectively. The upstream and downstream primers of GAPDH were 5'-CAGGAGGCATTGCTGATGAT-3' and 5'-GAAGGCTGGGGCATTT-3'.

### Cell transfection

3×10^5^ cells at the logarithmic growth stage were inoculated into a six-well cell culture plate. When the degree of cell fusion reached 50-70%, Lipofectamine 3000 was applied to transfect the GIGYF1 small interfering RNA-Homo-2566 (si-GIGYF1-1: 5'-GGGACGAAACUGCAGAGAATT-3', 5'-UUCUCUGCAGUUUCGUCCCTT-3') and GIGYF1 small interfering RNA-Homo-3401 (si-GIGYF1-2: 5'-GGACAUACCAAUUAACUCUTT-3', 5'-AGAGUUAAUUGGUAUGUCCTT-3'). The cells were transfected with the GIGYF1 small interfering RNA and the negative control (NC).

### Transwell migration assay

The cells in each group were digested, and the medium was removed by centrifugation after digestion. Complete medium (600μL) was added to the lower chamber of the 24-well plate. The cells were re-suspended in serum-free medium, and 2×10^4^ cells were added to the upper chamber after counting, cultured at 37℃, 5% CO_2_ for 24-48h, fixed with 4% paraformaldehyde at room temperature for 30 minutes, and stained with crystal violet for 15 minutes. After cleaning with PBS buffer, the cells in the upper compartment were wiped with sterile cotton swabs. Five fields were selected randomly under a microscope, and images were collected to calculate the number of transferred membrane cells.

### Wound healing assay

4.5×10^5^ cells at the logarithmic growth stage were inoculated onto 6-well plates for further culture, then transfected and cultured. Then, draw a line with the tip of the 10µL pipette and wash with PBS. Add FBS free medium for cell culture, and observe the degree of cell healing under the microscope.

### Colony-formation assay

After transfection, 1×10^3^ cells were inoculated onto 6-well plates for further culture, and the medium was changed every 3 days. Cells were incubated for 10-14 days, fixed with 4% paraformaldehyde, and stained with crystal violet.

### Cell proliferation assay

After transfection, 1×10^3^ cells were inoculated onto 96-well plates for further culture. Cell proliferation was analyzed by Cell Counting Kit-8 (cck-8). The microplate reader was thereby adopted for detecting absorbance value at 450 nm every 24 hours.

### Flow cytometry

The cell culture medium was sucked out into a suitable centrifuge tube first, the cells were then washed with PBS and digested with trypsin cell digestion solution without EDTA. After digesting the cells, the collected cell culture medium was added, mixed slightly, and transferred to the centrifuge tube. The supernatant was discarded after centrifugation at 1000g for 5 minutes, and the cells were collected. The cells were gently resuspended with PBS and counted. The suspended cells (1-5×10^5^) were centrifuged at 1000g for 5 minutes. The supernatant was then discarded, and 500µL of a binding solution was added to suspend the cells gently. Subsequently, 5µL of Annexin V-FITC was added and mixed gently, and 5µL of propidium iodide (PI) was then added and mixed gently. The resulting mixture was then incubated at room temperature, away from light, for 10 minutes. BD FACS Calibur flow cytometer (Becton-Dickinson, Franklin Lakes, NJ, USA) was utilized for cell analysis.

### Immunofluorescence assay

The cells were transfected with the GIGYF1 small interfering RNA for 48h, the cells were digested and counted, and 1×10^4^ cells were inoculated on the cell slides. After fixation with 4% paraformaldehyde, the cell membrane was infiltrated with 0.5% Triton X-100 and blocked with 5% bovine serum albumin. The primary antibody was incubated overnight at 4℃, cleaned with PBS, incubated with GFP-labeled goat anti-rabbit IgG, cleaned again with PBS, incubated with 1μg/ml Hoechst 33342. The cell slides were removed, sealed with an anti-fluorescence quencher and kept away from light. Finally, the cells were observed by laser confocal scanning microscope.

### Observation of autophagosomes by laser confocal microscopy

The cells were transfected with the pcDNA-eGFP-LC3 plasmid and treated with rapamycin for 24h, the cells were digested and counted, and 1×10^4^ cells were inoculated on the cell slides. The next day, the cells were fixed with 4% paraformaldehyde and washed with PBS. The cells were then stained with Hoechst 33342 and washed with PBS. The cell slides were removed, sealed with an anti-fluorescence quencher and kept away from light. Finally, the cells were observed by laser confocal scanning microscope.

### Western Blotting

The cells were washed two or three times with ice-cold PBS and lysed in RIPA buffer containing protease and phosphatase inhibitors. The total protein in the supernatant was measured using a bicinchoninic acid (BCA) protein assay kit. Equal amounts of protein were resolved by 10% SDS-PAGE gels and transferred to PVDF membranes electrophoretically. The membranes were blocked with 5% nonfat dry milk powder and then incubated overnight with the primary antibodies at 4 °C. Washing with TBST at room temperature, incubating with appropriate secondary antibody, washing again with TBST and then exposing using enhanced chemiluminescence (ECL) system according to the manufacturer's instructions.

### Statistical Analysis

Statistical analysis of the data was performed using GraphPadPrism 8.0.2 software. ImageJ software was used for Image processing and analysis, including cell counting, Western blotting quantitative analysis, fluorescence intensity analysis. The data are expressed as X ± S. A t-test was used for pairwise comparison between groups, and the assay was repeated three times. Univariate analysis of the variance (ANOVA) was used to compare the groups, and an LSD t-test was used for pairwise comparison. The P-value <0.05 was considered significant.

## Results

### GIGYF1 is highly expressed in GC tissue

**Figure 1:**
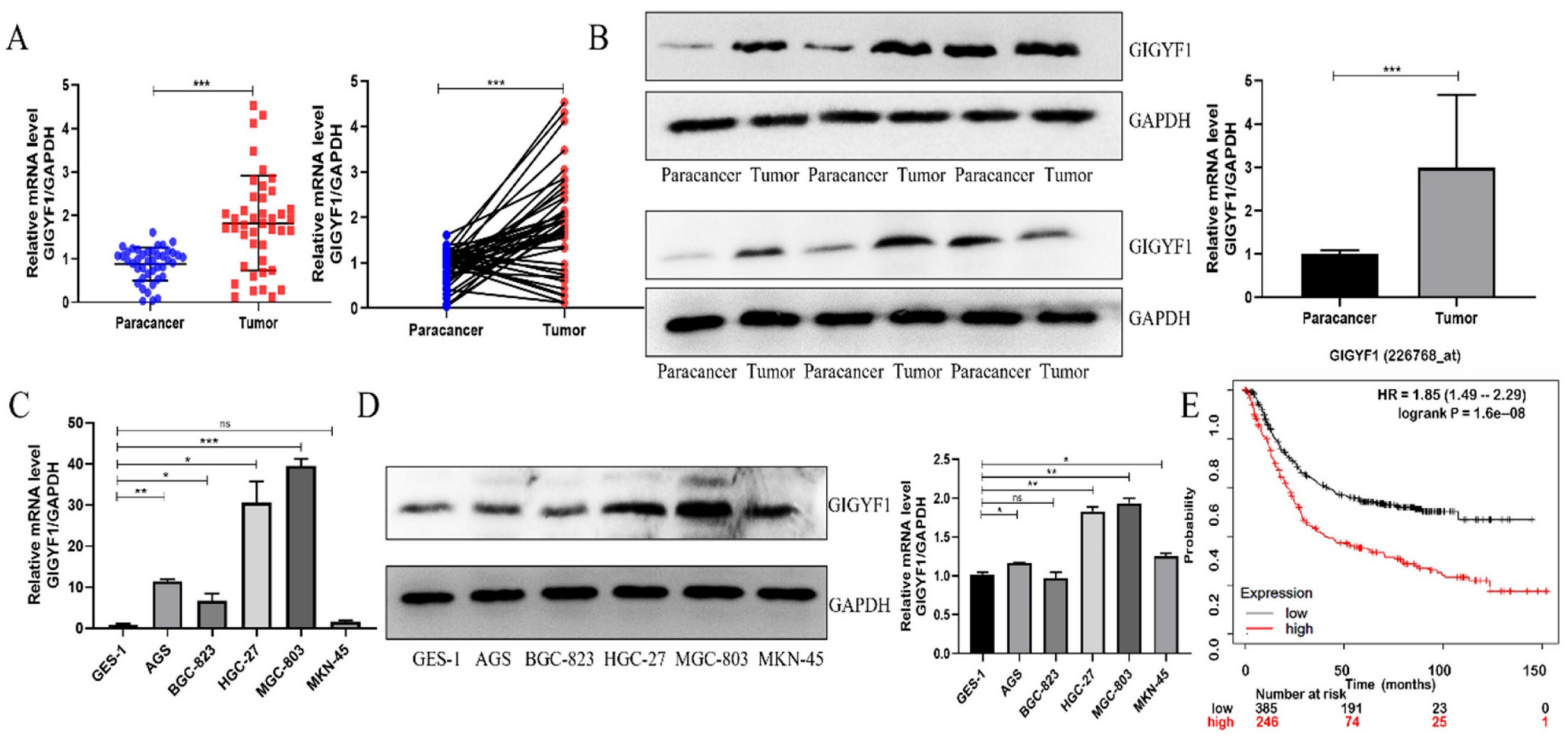
The expression of GIGYF1 in GC tumor tissue samples is higher than that in paracancer tissue samples, and high expression of GIGYF1 means poor prognosis. (A) Relative mRNA level of GIGYF1 in 42 pairs of fresh GC tumor tissue samples and paracancer tissue samples. (B) Relative protein level of GIGYF1 in fresh GC tumor tissue samples and paracancer tissue samples. (C) Detection of GIGYF1 expression in GC cell lines by qRT-PCR. (D) Detection of GIGYF1 expression in GC cell lines by western blotting. (E) The survival analysis of GIGYF1 expression in GC patients was analyzed with Kaplan Meier plotter. The high expression of GIGYF1 in GC patients showed poor prognosis. *p < 0.05, **p < 0.01, ***p < 0.001.

We collected 42 pairs of GC patients' tumor tissue samples and paracancer tissue samples, and detected the expression of GIGYF1 in the tissue samples by real-time fluorescent quantitative PCR. The results showed that the expression of GIGYF1 in 31 of the 42 (15/22; 73.81%) pairs of tissue samples was higher than that in the paracancer tissue samples (Fig. 1A), which was related to tumor size and TNM stage (Table 1). The expression level of GIGYF1 protein in 12 pairs of tumor tissue samples and paracancer tissue samples was detected by western blotting, and proved that the expression level of GIGYF1 protein in tumor tissue samples was generally higher than that in paracancer tissue samples (Fig. 1B). The mRNA expression of GIGYF1 in GC cell lines was detected by qRT-PCR and western blotting. The results showed that GIGYF1 was highly expressed in human GC cell lines compared with the human normal gastric epithelial cells, and the highest expression was in MGC-803 cells (Fig. 1C,1D). Kaplan-Meier plotter (http://www.kmplot.com) database evaluated the relationship between GIGYF1 mRNA expression level and prognosis in GC patients^17^. The results showed that a high level of GIGYF1 mRNA expression implied a poor prognosis (P=1.6e-8) (Fig. 1E).

### Low expression of GIGYF1 inhibits cell metastasis and EMT process

**Figure 2:**
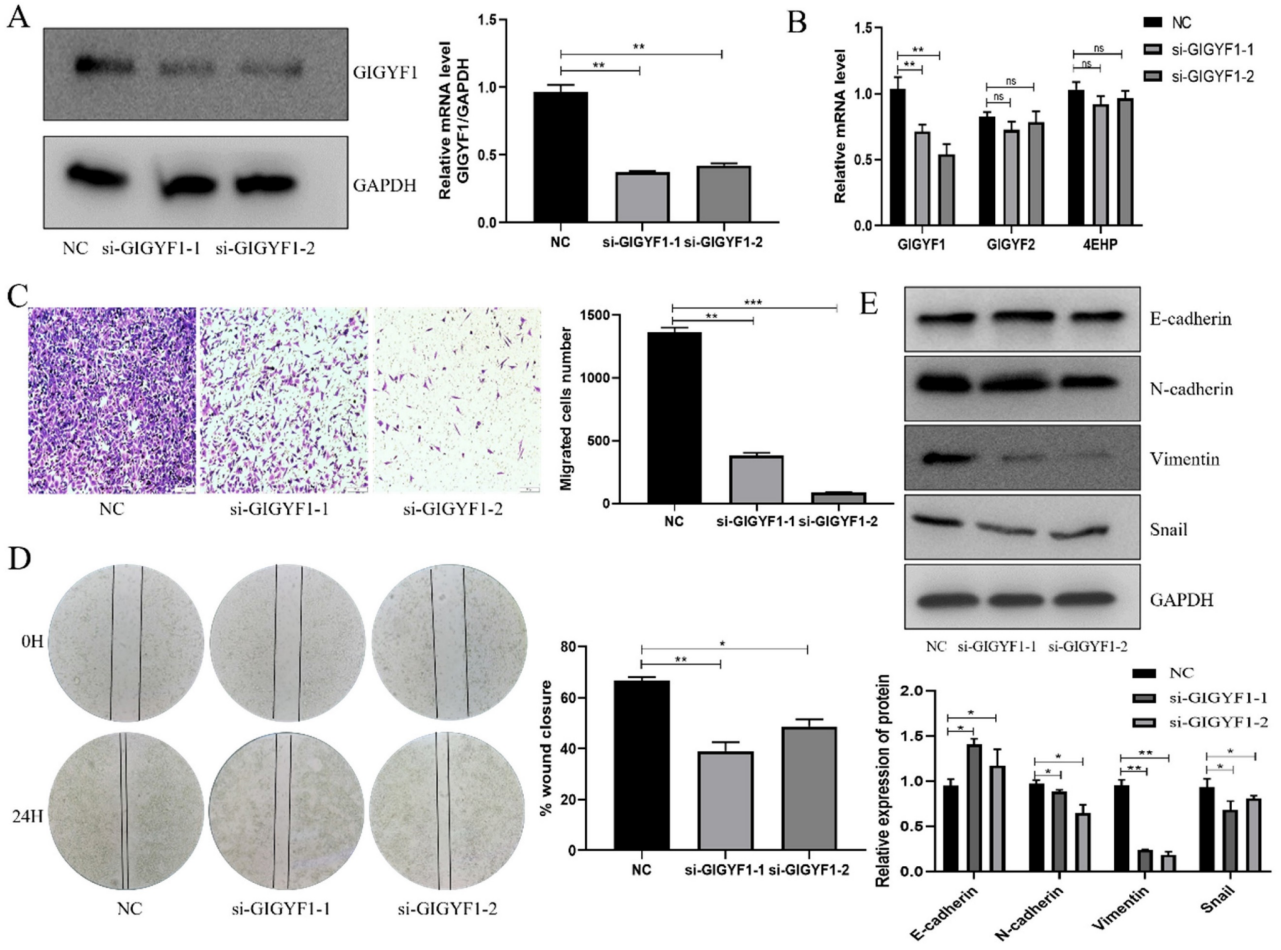
Low expression of GIGYF1 inhibits cell metastasis and EMT process. (A) Knockdown GIGYF1 with small interfering RNA in MGC-803 cells. (B) The changes in the expression of GIGYF2,4EHP after knocking down GIGYF1. (C) Transwell assays to detect cell migration. (D) Detection of cell healing ability by wound healing assays. (E) Western blotting detected the change of EMT related protein expression after GIGYF1 knockdown. *p < 0.05, **p < 0.01, ***p < 0.001.

In MGC-803 cells, small interfering RNA was transfected to knock down the expression of GIGYF1, and the knock down efficiency was detected by western blotting and qRT-PCR (Fig. 2A, 2B). The expression levels of GIGYF2, and 4EHP at the mRNA level were detected by qRT-PCR after knockdown of GIGYF1. The experiment showed that the expression level of GIGYF1 decreased, while there was no significant change in GIGYF2, indicating that the knock-down of GIGYF1 was specific. At the same time, the expression level of 4EHP did not change significantly, indicating that the expression of endogenous 4EHP was not affected after GIGYF1 was knocked down, but it must be combined with GIGYF1 as a complex to play its role (Fig. 2B). Transwell assays proved that after knockdown of GIGYF1 gene, the transfer ability of cells decreased significantly (Fig. 2C), and wound healing assays proved that the healing ability of cells also decreased (Fig. 2D). Western blotting showed that the expression of E-cadherin protein increased and the expression of N-cadherin, Vimentin and Snail protein decreased after knockdown of GIGYF1, indicating that the decrease of GIGYF1 expression inhibited the EMT process of cells (Fig. 2E).

### Low expression of GIGYF1 inhibits cell proliferation, promotes cell apoptosis and autophagy

**Figure 3:**
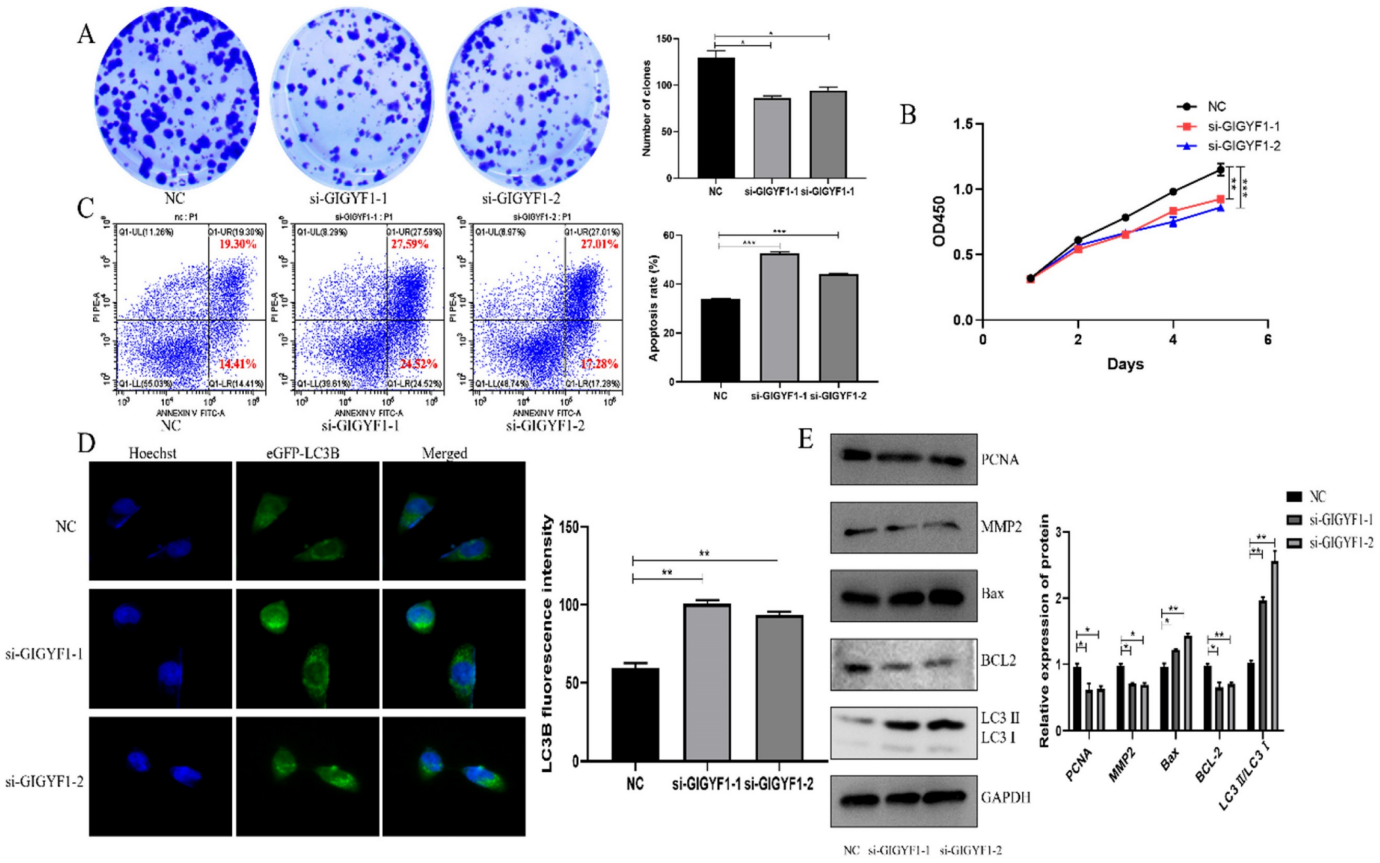
Low expression of GIGYF1 inhibits cell proliferation, promotes cell apoptosis and autophagy. (A) Effect of low expression of GIGYF1 on cell cloning detected by plate cloning test. (B) The value of OD450 was detected by cck-8 assay. (C) Detection of apoptosis rate by flow cytometry. (D) Detection of autophagosome formation by laser confocal scanning. (E) Western blotting was used to detect the protein expression of proliferation, apoptosis and autophagy. *p < 0.05, **p < 0.01, ***p < 0.001.

After knockdown of GIGYF1 gene in MGC-803 cells, the number and volume of cell clones decreased significantly, indicating that the cell proliferation capacity decreased (Fig. 3A); the OD450 value detected by cck-8 assay decreased significantly, indicating that the cell activity was weakened (Fig. 3B); the apoptosis rate of cells was significantly increased, indicating that the apoptosis of cells was promoted (Fig. 3C); the number of autophagosomes increased significantly, indicating that the autophagy was promoted (Fig. 3D). Western blotting showed that the expression of MMP2 and PCNA decreased; Bax protein expression increased and BCL-2 protein expression decreased; The expression of LC3 Ⅱ/LC3 Ⅰ increased (Fig. 3E).

### Low expression of GIGYF1 inhibits the ERK and AKT pathways

**Figure 4:**
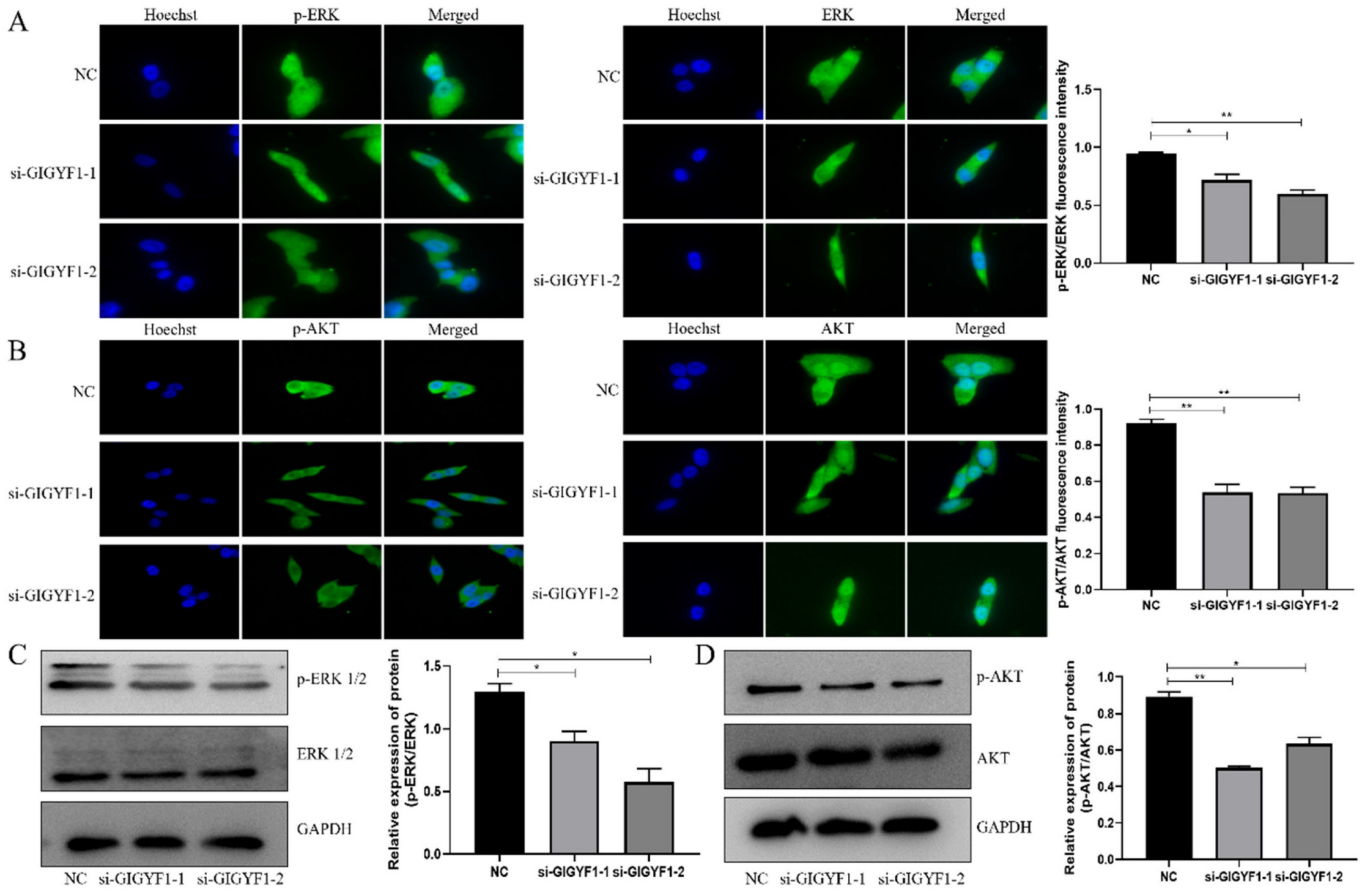
Low expression of GIGYF1 inhibits the ERK and AKT pathways. (A) Detection of p-ERK and ERK expression in cells by immunofluorescence assay. (B) Detection of p-AKT and AKT expression in cells by immunofluorescence assay. (C) Detection of p-ERK and ERK protein expression by western blotting. (D) Detection of p-AKT and AKT protein expression by western blotting. *p < 0.05, **p < 0.01, ***p < 0.001.

The immunofluorescence assay showed the expression of p-ERK/ERK and p-AKT/AKT in cells decreased significantly after knockdown of GIGYF1 gene (Fig. 4A, B), and the western blotting also detected the expression of p-ERK/ERK and p-AKT/AKT protein decreased significantly (Fig. 4C, D).

## Discussion

GC is a malignant tumor with multiple factors, multiple genes and multistage development^1^,^18^. Early symptoms are not typical, and radical treatment can be obtained under endoscopy. However, most patients are diagnosed in the middle and late stages^3^,^19^. Progressive GC is mainly surgery combined with chemotherapy, but patients with GC are prone to relapse or metastasis after surgery, and long-term chemotherapy has greater toxicity^1^,^20^-^21^. Therefore, we need to screen out early diagnostic markers with higher sensitivity.

GIGYF1 gene has been proved to play an important role in the occurrence and progression of many diseases, such as autism, type 2 diabetes^9^,^22^-^25^. However, whether it plays a role in the occurrence and progression of gastric cancer has not been confirmed.

The experimental results showed that GIGYF1 gene was highly expressed in GC tumor tissue and was related to tumor size and TNM stage. High GIGYF1 expression meant poor prognosis.

Compared with GES-1 cells, GIGYF1 gene is highly expressed in GC cell lines. After GIGYF1 gene was knocked down by small interfering RNA in MGC-803 cells, there was no significant change in GIGYF2, indicating that the knock-down of GIGYF1 was specific. At the same time, the expression level of 4EHP did not change significantly, indicating that the expression of endogenous 4EHP was not affected after GIGYF1 was knocked down, but it must be combined with GIGYF1 as a complex to play its role^26^-^27^. After GIGYF1 knockdown, cell migration, scratch healing ability and EMT process were significantly inhibited, and the formation of clone and the rate of cell proliferation were significantly decreased, accompanied by changes in related proteins, indicating that the low expression of GIGYF1 inhibited cell metastasis and proliferation. The apoptosis rate and the formation of autophagosome were significantly increased, and related proteins were also changed, indicating that low expression of GIGYF1 promoted apoptosis and autophagy of cells. Meanwhile, the expression of p-ERK/ERK and p-AKT/AKT decreased significantly. Therefore, low expression of GIGYF1 inhibits cell metastasis, proliferation, promotes cell apoptosis and autophagy, during which the ERK and AKT signaling pathways are inhibited.

In conclusion, GIGYF1 gene is highly expressed in GC tumor tissue, and low expression of GIGYF1 can inhibit the occurrence and progression of GC. Therefore, GIGYF1 gene is expected to become a biomarker and therapeutic target of GC.

## Figures and Tables

**Table 1:** The relationship between the expression level of GIGYF1 and the clinical characteristics of GC

Parameters	No. of patients (n=42)	High expression of GIGYF1 (n=31)	Low expression of GIGYF1 (n=11)	P value
Gender				0.3717
Male	31	24	7	
Female	11	7	4	
Age (years)				0.7586
>65	29	21	8	
≤65	13	10	3	
Tumor size (cm)				0.0131*
>4	28	24	4	
≤4	14	7	7	
Differentiation				0.5055
Poor	30	23	7	
Well-moderate	12	8	4	
TNM stage				0.0029**
I + II	15	7	8	
III + IV	27	24	3	

Bold values represent statistically significant P values (*p < 0.05, **p < 0.01, ***p < 0.001).
